# Weight change in the first 30 days among infants born less than 2000 grams in Guinea-Bissau and Uganda

**DOI:** 10.1038/s41598-024-61898-3

**Published:** 2024-05-14

**Authors:** Raimundo Co, Victoria Nankabirwa, Carlito Bale, Augusto Braima de Sa, Susanne P. Martin-Herz, Emily Blair, Lance Pollack, Victoria Laleau, Valerie Flaherman, Amy Sarah Ginsburg

**Affiliations:** 1 International Partnership for Human Development, Bissau, Guinea-Bissau; 2 Makerere University School of Public Health, Kampala, Uganda; 3 University of California San Francisco, San Francisco, CA USA; 4 Clinical Trials Center, University of Washington, Building 29, Suite 250, 6200 NE 74th Street, Seattle, WA 98115 USA

**Keywords:** Newborn, Low birth weight, Underweight, Growth, Low- and middle-income countries, Health care, Medical research

## Abstract

Despite the high prevalence of low birth weight infants in sub-Saharan Africa and the associated poor outcomes, weight change during the newborn period has not been well characterized for this population. We prospectively assessed growth over the first 30 days among 120 infants born < 2000 g (g) in Guinea‐Bissau and Uganda, and compared it to a similar cohort of 420 infants born ≥ 2000 g. Among those born < 2000 g, mean birth weight was 1747 ± 164 g, and initial weight loss was 8.25 ± 4.40% of birth weight prior to the initiation of weight gain at a median of 3 (interquartile range 2, 4) days of age. This initial weight loss was more pronounced (8.25 vs 6.06%; *p* < 0.001) and lasted longer (median 3 vs 2 days; *p* < 0.001) than for infants born ≥ 2000 g. The initial period of weight loss was an important predictor of growth at 30 days in both cohorts. Infants born < 2000 g on average grew proportionately to their size at birth but did not experience catch-up growth; their weights at 30 days remained much lower than that of infants born ≥ 2000 g and most remained severely underweight. Targeted interventions to optimize early growth should be investigated.

## Introduction

As Africa experiences the fastest population growth globally, particularly in the number of births and children, sub-Saharan Africa also experiences the greatest gaps in healthcare.^1–3^ Low birth weight (LBW) newborns are especially vulnerable, and inadequate newborn growth is associated with increased risks of neurodevelopmental and immunologic impairment and susceptibility to infections.^4–7^ The first month of life is key to establishing healthy growth. Data show that during the newborn period, healthy U.S. infants typically lose weight and then begin growth at about 3 days of age, increasing their weight about 30% by 30 days of age.^8–11^ However, similar data on early weight loss, early weight gain, and growth trajectory over the first 30 days are lacking for the vulnerable population of LBW infants in sub-Saharan Africa, where a high prevalence of preterm birth may have a deleterious influence on infant growth trajectory.^12^.

Assessing outcomes of LBW infants in Uganda, one study reported that at 21 days of age, only 52% had regained their birth weight.^13^ A study of moderately LBW infants born 1500–2499 g (g) in India, Malawi, and Tanzania showed that this LBW population of infants appears to grow slowly and be at much higher risk of long-term growth impairment, but did not examine the relationship between initial weight loss, initial weight gain, and subsequent growth impairment. This study also reported that growth outcomes of LBW infants varied with gestational age at birth. Our team investigated patterns of initial weight loss, initial weight gain, and subsequent growth impairment in Guinea-Bissau and Uganda among infants born ≥ 2000 g and found that initial weight loss occurred for a median of 2 days following birth, with weight then increasing by 33% by 30 days of age.^14^ However, these patterns have not been previously examined in smaller LBW infants (< 2000 g) in Africa. It is unclear whether these smaller LBW infants experience similar growth trajectories as larger infants (≥ 2000 g), and whether they begin to gain weight trending toward norms for age in the first 30 days of life in the absence of a targeted intervention.

## Methods

At health facilities in Guinea-Bissau and Uganda, we enrolled 120 singleton infants with birth weights < 2000 g between March 2020 and January 2021 and defined this as our primary cohort. For this primary cohort, birth weight was defined as weight measured by trained study staff in infants < 6 h of age. Infants were eligible for enrollment if their mothers were at least 18 years old and intended to breastfeed for at least 6 months. In Guinea-Bissau, enrollment occurred at Simao Mendes Hospital in Bissau, and in Uganda, enrollment occurred at Mukono Health Center in Mukono, and Kitebi and Kawaala Health Centers in Kampala. We excluded infants with respiratory distress, World Health Organization newborn danger signs,^15^ major congenital anomalies, or maternal or infant contraindications to breastfeeding. We used a convenience sampling strategy for selection of enrollment sites and infants.

Trained study staff recruited, screened, and enrolled mothers and infants, and written informed consent was obtained from the mother for herself and her infant. Using a standardized protocol, trained study staff obtained duplicate weights and lengths for naked enrolled infants within 6 h of birth and at 1, 2, 3, 4, 5, 12, and 30 days of age, with a Seca 334 scale (Seca Inc., Wandsbek, Germany) accurate to ± 5 g and stadiometer (Seca Inc., and Pelstar, LLC), respectively; two additional measurements were taken if the initial two measurements varied by 15 g or by 0.5 cm (cm), respectively, for weight and length.

Intention to breastfeed was assessed at enrollment as an inclusion criterion. Infant dietary intake including breastfeeding and any supplementary feeding was assessed at enrollment and subsequent study visits using an instrument previously validated for breastfeeding infants in the first week of life in low- and middle-income countries.^16^ Enrolled mothers were surveyed regarding covariates related to enrolled infant growth, including maternal age, educational attainment, marital status, parity, location of delivery, and any infant adverse events including lethargy, convulsions, fever, low body temperature, difficulty breathing, vomiting, diarrhea, blood in stool, jaundice, and umbilical redness. Study visits occurred at the enrollment health facilities or during home visits as preferred by the participants. If necessary, participants were traced and located using provided maps and contact information. In this observational study, the study team did not have access to clinical data regarding change in infant weight, did not provide health care to enrolled infants, and encouraged mothers to access their usual sources of care. Referrals to medical care were made by the study team as needed. Travel reimbursement was provided; no other incentives were offered.

Our primary cohort was compared to a secondary cohort of 420 singleton infants with birth weights ≥ 2000 g who were enrolled between April and December 2019 and who were followed through 30 days of age using similar procedures. The secondary cohort had been enrolled at the same health facilities where the primary cohort was enrolled, and also at Bissora Hospital in Bissora (n = 10) and local village facilities in Guinea-Bissau (n = 11). Bivariate analyses assessed group differences in infant and maternal characteristics. Associations with dichotomous and continuous outcomes were tested using the chi-square statistic and unpaired student’s t-test, respectively. In cases where the equal variance assumption was not met, a *p*-value based on an adjusted t statistic and adjusted degrees of freedom was computed. All analyses were conducted in SPSS Version 22.0 (IBM Corp, Armonk, NY).

This study was approved by the UCSF Institutional Review Board, the Guinea-Bissau National Committee on Ethics in Health (Comite Nacional de Etica na Saude), the Higher Degrees, Research and Ethics Committee of Makerere University, and the Uganda National Council of Science and Technology. All study activities were performed in accordance with relevant guidelines and regulations.

## Results

In our primary cohort, a total of 120 infants, of whom 51 (42.5%) were female, were enrolled in Guinea-Bissau and Uganda. One newborn death occurred prior to 1 day of age, and a total of 16 deaths occurred prior to 30 days of age (10 in Guinea-Bissau and 6 in Uganda; *p* = 0.283). Male infants experienced higher mortality than female infants (14 vs 2 deaths; *p* = 0.009).

During the first day after birth, 14 (11.8%) infants in the primary cohort were not fed. Among the 105 (88.2%) infants who were fed, 101 (96.2%) were breastfed (97 (92.4%) exclusively), and 8 (7.6%) received only a liquid supplement of tea or sweetened, herbal or flavored water. No enrolled infant received formula the day after birth. At 30 days of age, 101 (out of 120, 84.2%) infants were available for follow-up; all were breastfeeding, while 11 (10.9%) also were receiving supplementary feeding. Among those supplemented at 30 days of age, formula was received by 3, plain water by 3, cow’s milk by 2, sugarcane juice by 1, tomato soup by 1, and herbal water by 2.

In this primary cohort enrolled with convenience sampling, baseline characteristics such as maternal education, marital status, household size, primiparity, and infant weight and length differed by country of enrolling facility (Table 1). Infant adverse events in the first 30 days, which occurred for 34 (28.3%) infants also differed by country of enrolling facility (7 among infants born at enrolling facilities in Guinea-Bissau and 27 among infants born at enrolling facilities in Uganda; *p* < 0.001). Overall mean birth weight in this cohort was 1747 ± 164 g and overall mean birth length was 41.1 ± 2.2 cm, with birth length 1.4 cm higher in Guinea-Bissau than Uganda (p = 0.001); median birth weight was 1761.25 g with an interquartile range (IQR) 1630.00—1881.88 g. Only 5 of the 120 infants (4.2%) had birth weights below 1500 g. Significant differences in these baseline characteristics were also noted between the primary and secondary cohorts. (Table 1).

**Table 1:** Baseline infant and maternal characteristics.

	Comparisons within primary cohort			Comparisons between cohorts		
	Guinea-Bissau	Uganda	Guinea-Bissau vs Uganda *p*-value	Primary cohort (< 2000 g at birth)	Secondary cohort (> 2000 g at birth)	Primary vs secondary *p*-value
Cohort size (Number)	60	60		120	420	
Infant mortality by day 30, n (%)	10 (16.7)	6 (10.0)	0.283	16 (13.3)	2 (0.5)	** < 0.001**
Infant characteristics						
Male sex, n (%)	33 (55.0)	36 (60.0)	0.580	69 (57.5)	207 (49.3)	0.112
Cesarean delivery, n (%)	10 (16.7)	19 (31.7)	0.055	29 (24.2)	5 (1.2)	** < 0.001**
Age (hours) at birth weight, median (IQR)	1.58 (1.10–3.50)	3.55 (3.00–4.38)	** < 0.001**	3.00 (1.47–4.08)	3.09 (1.61–4.37)	0.417
Weight (g) at birth, mean (SD)	1727 (166.66)	1767 (159)	0.177	1747 (163)	3124 (422)	** < 0.001**
Length (cm) at birth, mean (SD)	41.82 (1.73)	40.47 (2.49)	**0.001**	41.14 (2.24)	48.55 (2.11)	** < 0.001**
MUAC (cm) at birth, mean (SD)	7.57 (0.53)	8.84 (0.96)	** < 0.001**	8.19 (1.00)	10.63 (0.93)	** < 0.001**
Maternal characteristics						
Age (years), mean (SD)	26.65 (6.84)	25.32 (6.11)	0.296	25.90 (6.44)	25.99 (5.37)	0.899
Weight (kg), mean (SD)*	59.2 (11.3)	58.5 (9.2)	0.726	58.9 (10.2)	62.4 (12.4)	**0.008**
Height (cm), mean (SD)*	158.4 (4.5)	157.5 (6.9)	0.453	158.0 (5.9)	158.4 (5.9)	0.463
BMI, mean (SD)*	23.55 (4.02)	23.53 (3.09)	0.972	23.54 (3.54)	24.66 (3.98)	**0.010**
BMI < 21, n (%)*	14 (29.8)	12 (22.6)	0.416	26 (26.0)	71 (18.1)	0.075
Married, n (%)	29 (61.7)	51 (86.4)	**0.003**	80 (75.5)	246 (60.6)	**0.005**
Education (years), mean (SD)	7.48 (4.37)	10.44 (2.78)	** < 0.001**	9.14 (3.84)	7.61 (4.39)	**0.001**

n, number; %, percent; IQR, interquartile range; SD, standard deviation; g, gram(s); kg, kilogram(s); cm, centimeter(s); MUAC, mid-upper arm circumference; BMI, body mass index.

Note: Male vs female comparisons within primary cohort for infant weight, length, and MUAC were all non-significant.

*Measured at 30 days postpartum to account for peripartum weight fluctuations.

Bolded p-values are statistically significant.

At 30 days of age, mean weight in the primary cohort was 2333 ± 452 g and mean length was 44.9 ± 2.2 cm. Mean weight change and mean length change over the first 30 days were 571 ± 375 g (34 ± 21% above birth weight), and 3.7 ± 2.1 cm (9.2 ± 5.5% above birth length), respectively. Of those who survived to 30 days of age, fifty (49%) received inpatient care other than routine postpartum care in the first 30 days. The overall prevalence of being severely underweight at 30 days of age (male infants < 2.9 kg (kg) and female infants < 2.7 kg) was 80%. Among the 82 infants who were severely underweight at 30 days, birth weight ranged from 1465 to 1990 g for males and 1310–1995 g for females. Among the 20 infants who were not severely underweight at 30 days, birth weight ranged from 1703 to 1995 g for males and 1773–1993 g for females. Of note, at 30 days of age, 3 (2.9%) infants weighed less than at birth. Early weight loss and timing of weight nadir had a stronger association with the outcome of being severely underweight at 30 days than did baseline variables (Table 2).

**Table 2:** Infant and maternal characteristics by status of severely underweight at 30 days of age.

		Severely underweight at 30 days of age		
		No	Yes	No vs yes *p*-value
Number (%)		20 (19.6)	82 (80.4)	
Site, n (%)	Guinea-Bissau	7 (14.6)	41 (85.4)	0.228
	Uganda	13 (24.1)	41 (75.9)	
Infant characteristics				
Infant’s sex, n (%)	Male	9 (17.0)	44 (83.0)	0.487
	Female	11 (22.4)	38 (77.6)	
Method of delivery, n (%)	Vaginal	15 (19.5)	62 (80.5)	1.000
	Cesarean	5 (20.0)	20 (80.0)	
Infant’s weight at birth in grams, mean (SD)		1890 (89)	1730 (145)	** < 0.001**
Percent of birth weight lost at weight nadir, mean (SD)		-5.43 (2.83)	-8.93 (4.46)	** < 0.001**
Infant’s length at birth in cm, mean (SD)		42.55 (1.87)	40.88 (2.06)	**0.001**
Infant’s mid-upper arm circumference at birth in cm, mean (SD)		8.47 (0.72)	8.26 (1.07)	0.409
Adverse events through day 30, n (%)	Any	10 (30.3)	23 (69.7)	0.060
	None	10 (14.5)	59 (85.5)	
Maternal characteristics				
Mother’s age in years, mean (SD)		23.75 (5.64)	26.33 (6.48)	0.107
Mother’s education in years, mean (SD)		9.65 (2.54)	8.83 (4.11)	0.264
Mother’s marital status, n (%)	Married	15 (19.7)	61 (80.3)	1.000
	Not married	5 (20.0)	20 (80.0)	

n, number; %, percent; SD, standard deviation.

* Male infants < 2900 g and female infants < 2700 g at 30 days of age were defined as severely underweight in alignment with World Health Organization Child Growth Standards.

Bolded p-values are statistically significant.

Weight loss occurred prior to weight gain for all infants in the primary and almost all infants in the secondary cohorts. Overall, infants in the primary cohort lost 8.2 ± 4.4% of their birth weight before weight gain began at a median of 3 (interquartile range 2, 4) days after birth. Infants in the primary cohort who remained below 2000 g at 30 days of age experienced their weight nadir at 5.6 ± 6.0 days, while those whose weight at 30 days exceeded 2000 g experienced their weight nadir at 3.0 ± 1.8 days (p = 0.045). Infants in the primary cohort who remained below 2000 g at 30 days of age had their weight nadir 10 ± 4.6% below their birth weight, while those whose weight exceeded 2000 g at 30 days had their weight nadir 7.7 ± 4.2% below their birth weight (*p* = 0.025). Absolute weight change over the first 30 days differed substantially between the primary and secondary cohorts (Table 3).

**Table 3:** Weight change from Day 0 to Day 30.

	Raw weight change [mean (SD)]			Percent weight change [mean (SD)]		
	Primary cohort < 2000 g (N = 104)	Secondary cohort > 2000 g (N = 405)	Primary vs secondary *p*-value	Primary cohort < 2000 g (N = 104)	Secondary cohort > 2000 g (N = 405)	Primary vs secondary *p*-value
*Mean change in weight from birth to…*						
Day 1	− 60 (34)	− 131 (75)	** < 0.001**	− 3.42 (1.95)	− 4.18 (2.32)	**0.002**
Day 2	− 108 (57)	− 157.97 (114.12)	** < 0.001**	− 6.17 (3.13)	− 5.02 (3.52)	**0.003**
Day 3	− 116 (80)	− 99 (135)	0.107	− 6.65 (4.52)	− 3.16 (4.29)	** < 0.001**
Day 4	− 100 (98)	− 44 (140)	** < 0.001**	− 5.73 (5.55)	− 1.37 (4.42)	** < 0.001**
Day 5	− 81 (110)	+ 11 (156)	** < 0.001**	− 4.70 (6.27)	+ 0.42 (5.03)	** < 0.001**
Day 12	+ 39 (166)	+ 303 (231)	** < 0.001**	+ 2.04 (9.24)	+ 9.79 (7.52)	** < 0.001**
Day 30	+ 570 (375)	+ 1000 (342)	** < 0.001**	+ 31.98 (20.35)	+ 32.92 (12.20)	0.660
Weight nadir	− 144 (77)	− 190 (119)	** < 0.001**	− 8.25 (4.40)	− 6.06 (3.65)	** < 0.001**
Day of weight nadir* [mean (SD)]	3.57 (3.45)	2.09 (2.12)	** < 0.001**	–	–	–

SD, standard deviation; g, gram(s); N, number.

* Median (interquartile range): small infants 3 (2–4); larger infants 2 (1–2).

Bolded p-values are statistically significant.

## Discussion

In this primary cohort of smaller LBW infants born 1210 to 1995 g in Guinea-Bissau and Uganda without respiratory distress, danger signs, or major congenital anomalies, an average of 8.25% of birth weight was lost prior to initiating weight gain at a median of 3 (IQR 2, 4) days of age. This initial weight loss was more pronounced (8.25 vs 6.06%; *p* < 0.001) and lasted longer (3 vs 2 days) than a similar secondary cohort of infants with birth weights ≥ 2000 g at the same sites.^14^ Initial weight loss among primary cohort infants was highly associated with poor growth at 30 days of age, as was age at which weight loss ended and weight gain was initiated. These results are consistent with findings in the existing literature showing that infants born LBW in sub-Saharan Africa are at high risk of ongoing growth impairment, and also reveal that pronounced initial weight loss may be a factor contributing to long-term outcomes for these children.^17–19^ Importantly, our results also showed that many infants did not receive either breast milk or artificial milk on the day after birth. Taken together, these findings suggest that improved strategies for infant feeding early in the newborn period might have important potential to improve and reduce the risk of growth impairment in this population.

Our study had several limitations, including that validated assessment of gestational age was not available, and Cesarean births were rare, so we were not able to report how newborn growth varied by gestational age or delivery method and were not able to calculate z-scores based on gestational age. Given that we enrolled only infants < 2000 g for this study and that the third centile for infants 37 weeks gestation was 2159 g for girls and 2260 g for boys, it is highly likely that most infants in this cohort were born preterm (< 37 weeks gestation); however, no testing was done to assess this. We also did not collect more detailed information on maternal pregnancy or delivery history, factors which may have impacted breastfeeding initiation. This is an important limitation because preterm birth influences supplementary feeding practices, time to weight nadir, and growth trajectory in the first 30 days.^20,21^ Our study used convenience sampling to enroll 120 infants in two countries and may not be representative of infant growth in either country or elsewhere. Although 96% of infants were breastfed, we did not collect detailed breastfeeding data and are not able to report on the role of latch score or duration and frequency of breastfeeding in this cohort of infants. While we used a carefully standardized method for weight measurement, some measurement error likely occurred, and this may impact the validity of our results. Of note, however, measurement error tends to bias results to the null, while many of our identified effect size estimates were large, suggesting that the study’s conclusions are likely sound despite this limitation.

Since our results show that early weight loss and slower initiation of weight gain are associated with worse outcomes, it is possible that smaller low birth weight infants (< 2000 g) may benefit from intervening early with targeted nutritional support in the first few days after birth to mitigate the difference between their weight loss and that of larger infants. In preterm infants, the rate of catch-up growth can be associated with neonatal morbidities, including impaired neurodevelopment, and long-term health consequences such as increased risk of cardiovascular, renal, and metabolic morbidities in adult life,^22^ so careful investigation of possible therapeutic options is urgently needed.

## Data Availability

De-identified, individual-level data on variables listed in tables will be shared with other researchers after approval of the proposed research question(s) and a signed data use agreement. For all data requests, Dr. Valerie Flaherman should be contacted at her email address Valerie.Flaherman@ucsf.edu.
